# Efficient genetic improvement of orphan crops cannot follow the old path

**DOI:** 10.1038/s41467-023-44458-7

**Published:** 2024-01-08

**Keywords:** Agricultural genetics, Genetic techniques, Genetic variation, Genome

## Abstract

Orphan crops hold the potential to diversify our food systems. Considering their unique characteristics, our deep understanding of major crops, and the availability of modern genomic tools, taking a different research path from what major crops have gone through could accelerate the genetic improvement of orphan crops.

Feeding the increasing world population during a time of rapid climate change is an urgent matter referenced by multiple Sustainable Development Goals set forward by the United Nations (https://sdgs.un.org/goals). To address this problem, significant efforts have focused on breeding major crops with improved yield, quality, and resilience to biotic and abiotic stresses. These efforts are exemplified by the “Green Revolution” in the 1960s that featured the development of high-yield dwarf cultivars via conventional plant breeding techniques and the recent “Gene Revolution” aided by molecular breeding approaches^1^. The striking issue remains that the predicted crop productivity achieved by breeding major crops alone will not be able to meet future food demand^2^. It is therefore too risky to simply rely on the three staple cereal crops, namely rice, maize, and wheat, to provide more than half of the calories consumed by humans^3^. We urgently need to diversify our food systems.

Orphan crops offer a potential solution to this issue. They are also known as “underutilized”, “minor”, “neglected”, “indigenous”, or “niche” crops^4^. They include cereals, pseudo cereals, such as quinoa, grain amaranth, and buckwheat, as well as legumes, and root crops. These crops are often cultivated in limited areas in Africa, Latin America, and Asia, but can be crucial for the livelihood and nutrition of the inhabitants and play an important cultural role in local food systems. Orphan crops may harbor desirable traits that have been lost from major crops during domestication and breeding, including resilience to extreme climate conditions, high nutritional value, and high photosynthetic efficiency^4^. Revealing the genetic basis underlying these traits will assist the breeding of future major and orphan crops to enhance global food security. However, efforts focusing on and investing specifically in orphan crops have been substantially less than those dedicated to major crops^5^.

With the fast development of genomic tools, the accumulating knowledge gained from major crops, and, most importantly, scientists’ increasing enthusiasm for these “hidden treasures”, the time has come to accelerate research on genomics and genetic improvement of orphan crops. In this regard, we have seen an increasing number of reports on reference genomes, population genomics, genotyping platforms, and mark-trait association of orphan crops during the last decade^4,6^. To maximize the benefit, investigations into these crops should be performed not only by scientists in high-income countries and/or well-funded institutions but also from regions where orphan crops are actually grown^7–9^. Examples of these include the African Orphan Crops Consortium (http://africanorphancrops.org/), Crops of the Future (https://foundationfar.org/consortia/crops-of-the-future-collaborative/), and the research programs conducted in multiple research centers of Consultative Group on International Agricultural Research (CGIAR; https://www.cgiar.org/).

“Considering the unique characteristics of orphan crops and the availability of modern genomic tools, we have the opportunity to take a different path than that historically used for major crops”

Considering the unique characteristics of orphan crops and the availability of modern genomic tools, we have the opportunity to take a different path than that historically used for major crops, which could accelerate the genetic improvement of orphan crops.

Unlike major crops, germplasm collections for orphan crops are often limited and their characterization is preliminary^10^. In fact, most orphan crops are only grown in their country of origin and are facing the threat of extinction due to the adoption of major crops and urbanization^10^. In addition, many orphan crops and their wild relatives are difficult to cross or have hybridization barriers, and some have a long generation cycle. All of these factors restrict the application of conventional breeding techniques^10^, which have been extensively used by our ancestors and modern breeders for the domestication and improvement of major crops. There is no doubt that solving these problems by increasing germplasm collection and documentation, overcoming hybridization barriers, and shortening life cycles through basic research will promote the implementation of conventional breeding techniques in orphan crops. However, considering the urgent need to diversify our food systems, it may not be the most efficient way to improve orphan crops. Compared to major crops, orphan crops have a shorter breeding history^5^. While some conventional breeding techniques could be applied to orphan crops, this assumes that the desired germplasm is available and the breeding techniques are readily applicable to the target species. Unfortunately, these assumptions have not yet been proven for most orphan crops. Therefore, conventional breeding may not be the best strategy for the efficient genetic improvement of certain orphan crops.

Many orphan crops are evolutionarily close to some major crops^11^. Therefore, we can benefit from the knowledge gained by breeding major crops, including the identification of genes that control key agronomic traits and the implementation of advanced breeding methodologies. Accumulating evidences have suggested functional conservation of orthologous genes between major and orphan crops^11^. Instead of cloning orphan crop genes from scratch, mining and utilizing genes that have been identified from closely related major crops could accelerate their genetic improvement. Similarly, breeding techniques that are suitable for a major crop could likely be applied directly to closely related orphan crops. Therefore, wisely utilizing knowledge gained from major crops rather than engaging in time-consuming exploration and troubleshooting processes can help give a head start in the breeding of orphan crops.

Recent technological advances in the various omics fields, including genomics, transcriptomics, metabolomics, proteomics, and phenomics, offer plant geneticists and breeders new tools to reveal genes/alleles underlying important traits with a fraction of the cost and at an unprecedented rate. In addition, genome editing tools have proven effective for trait improvement in orphan crops^12^. Since many orphan crops have not been fully domesticated, lessons learned from the domestications of various major crops can serve as a guide and lead to faster improvement of orphan crops, including using the de novo domestication approach^12^. Speed breeding has been shown to be effective in multiple major crop species, but its application in orphan crops has just begun^13^. Considering these technological possibilities, choosing the latest tools and protocols that have proven effective in major crops is crucial for the success of orphan crop genetic improvement. For these reasons, orphan crops can harness their latecomers’ advantages over major crops by taking advantage of the lessons learned over centuries of research.

We are optimally positioned to modernize breeding for orphan crops and to mainstream them into our food production systems by taking a different path from that traversed by major crops. By charting this new path, we must remind ourselves of the geographical and cultural specificity of orphan crops and diversify breeding objectives and methods correspondingly. Teaming up with *Scientific Reports* and *npj Sustainable Agriculture*, we have launched the *Orphan Crop Genomics and Improvement* Collection (https://www.nature.com/collections/fcbbiicehd) to call for submissions on orphan crops genomics, genetics, and breeding.
